# Risk Factors for Adhesion-Related Readmission and Abdominal Reoperation after Gynecological Surgery: A Nationwide Cohort Study

**DOI:** 10.3390/jcm12041351

**Published:** 2023-02-08

**Authors:** Masja Toneman, Tjitske Groenveld, Pepijn Krielen, Angelo Hooker, Rudy de Wilde, Luz Angela Torres-de la Roche, Atillio Di Spiezio Sardo, Philippe Koninckx, Ying Cheong, Annemiek Nap, Harry van Goor, Pille Pargmae, Richard ten Broek

**Affiliations:** 1 Department of Surgery, Radboudumc, 6525 GA Nijmegen, The Netherlands; 2 Department of Obstetrics and Gynecology, Zaans Medical Center (ZMC), 1502 DV Zaandam, The Netherlands; 3 University Hospital for Gynecology, Carl von Ossietzky University, 26121 Oldenburg, Germany; 4 Department of Public Health, School of Medicine, University of Naples Federico II, 80131 Naples, Italy; 5 Department of Gynecology, Katholieke Universiteit Leuven, 3000 Leuven, Belgium; 6 Faculty of Medicine, University of Southampton, Southampton SO16 6YD, UK; 7 Complete Fertility Centre, Southampton SO16 5YA, UK; 8 Department of Gynecology, Radboudumc, 6525 GA Nijmegen, The Netherlands

**Keywords:** operative risks, adhesions, gynecological surgery

## Abstract

More than half of women in developed countries undergo surgery during their lifetime, putting them at risk of adhesion-related complications. Adhesion-related complications include small bowel obstruction, chronic (pelvic) pain, subfertility, and complications associated with adhesiolysis during reoperation. The aim of this study is to predict the risk for adhesion-related readmission and reoperation after gynecological surgery. A Scottish nationwide retrospective cohort study was conducted including all women undergoing a gynecological procedure as their initial abdominal or pelvic operation between 1 June 2009 and 30 June 2011, with a five-year follow-up. Prediction models for two- and five-year risk of adhesion-related readmission and reoperation were constructed and visualized using nomograms. To evaluate the reliability of the created prediction model, internal cross-validation was performed using bootstrap methods. During the study period, 18,452 women were operated on, and 2719 (14.7%) of them were readmitted for reasons possibly related to adhesions. A total of 2679 (14.5%) women underwent reoperation. Risk factors for adhesion-related readmission were younger age, malignancy as indication, intra-abdominal infection, previous radiotherapy, application of a mesh, and concomitant inflammatory bowel disease. Transvaginal surgery was associated with a lower risk of adhesion-related complications as compared to laparoscopic or open surgeries. The prediction model for both readmissions and reoperations had moderate predictive reliability (c-statistics 0.711 and 0.651). This study identified risk factors for adhesion-related morbidity. The constructed prediction models can guide the targeted use of adhesion prevention methods and preoperative patient information in decision-making.

## 1. Introduction

Over half of women in developed countries undergo abdominal surgery during their life, causing a risk of developing adhesion-related symptoms [1,2,3]. In the national Scottish registry, almost 10,000 and 25,000 women annually were registered to undergo, respectively, their first gynecological or abdominal operation, translating to a respective incidence of 3.15 and 9.06 per 1000 person-years [1]. Considering the Scottish life expectancy of 81.1 years, one in four and three in four women will thus undergo, respectively, gynecological or abdominal surgery [4]. This seems representative for Western Society since in the United States the risk of abdominal surgery was 57% in a post-mortem study [2]. 

After open abdominal surgery, adhesions develop in up to 90% of patients [5]. Following laparoscopic procedures, the incidence might be lower, i.e., 54% to 70% of patients [6]. Adhesions cause a life-long risk of complications, such as small bowel obstruction, chronic abdominal pain, female subfertility, and difficulties during reoperations. Considering the high incidence of abdominal and gynecological surgeries, the burden of adhesion-related complications in women is high. In gynecology, factors that might impact adhesion formation comprise endometriosis, previous radiotherapy, malignancy, concomitant abdominal inflammation, infection, and foreign bodies [7]. However, it is not known whether these risk factors also affect the incidence of clinically relevant outcomes [7]. Estimating the effect of these and other risk factors on relevant adhesion-related complications is important for informed consent procedures, decision-making, and the application of adhesion-prevention strategies in gynecological surgery [8]. 

The aim of this study, therefore, is to construct prediction models, for the risk of adhesion-related readmissions and reoperations after gynecological surgery, using the data from the Scottish registry.

## 2. Materials and Methods

### 2.1. Research Design and Data-Retrieval

Data from the Scottish Medical Record Linkage Database, held by the Scottish National Health Service (NHS Scotland), were used. This database contains annually validated data on all inpatient and day-case hospital admissions, excluding maternity-related and psychiatric admissions, as described in detail in [1]. All women having a first gynecological operation between June 2009 and June 2011, without a history of previous abdominal (including cesarean section) surgery were included. Patients were followed until December 2017, documenting migration data and deaths. All eligible women were included since data on admission and operation had no opt-out. The surgical approaches were classified as open, laparoscopic or transvaginal, using the Office of Population Censuses and Surveys Classification of Interventions and Procedures version 4 (OPCS-4) codes. The same OPCS-4 codes were used to classify subsequent reoperations. 

Similar to previous SCAR (The Surgical and Clinical Adhesions Research) studies, all readmissions were screened for their potential association with adhesions [8,9]. Based on *International Classification of Diseases*, Tenth Edition (ICD-10) codes, the association between readmission and adhesions was classified as either readmission directly related to adhesions (e.g., adhesiolysis, adhesive small bowel obstruction) or readmission possibly related to adhesions (e.g., unspecified small bowel obstruction). Readmissions unlikely to be related to adhesions were not included in the analysis. 

Subgroup analyses were performed by anatomical site, such as uterus, vagina, fallopian tubes, ovaries, combined gynecological, and combined other. A procedure was categorized as ‘combined gynecological’ when multiple anatomical sites of the female reproductive tract were involved, i.e., uterus, vagina, Fallopian tubes, or ovaries. Procedures involving both the female reproductive tract and other organs (e.g., colorectal) were classified as ‘combined other’. 

Specific subgroup analyses were performed for patients receiving a hysterectomy since this subgroup is large and more homogeneous. 

Risk factors, based on the literature and the opinion of an expert panel, were age, surgical approach, operation site, malignancy, fertility-enhancing surgery, intra-abdominal infection, history of abdominal radiotherapy, intraperitoneal mesh placement, inflammatory bowel disease (IBD), endometriosis, and adhesiolysis.

### 2.2. Statistical Analysis

Descriptive analytics were used to describe baseline characteristics. Open, laparoscopic, and transvaginal groups were compared by ANOVA for numerical and chi-square for categorical data.

Cox regression was used for the construction of the prediction models. Predictive factors were screened by univariate Cox regression and those found to be significant were used for multivariable analysis with a stepwise backward selection removing variables with a *p* value > 0.2. The results were used to calculate the incidence of repeat surgery and the hazard ratios (HR) with 95 percent confidence intervals (CI).

Using the results of Cox regression analysis, the prediction for 2- and 5-year risk of readmission (direct and possibly adhesion-related) and reoperation was calculated [10]. In the analysis of readmissions possibly related to adhesions, the incidence of first directly or possibly related readmissions was recorded. Results are visualized as a nomogram. A nomogram is a graphical visualization of this prediction, using a scoring system with a scale for graphical calculations. Points are attributed for each prognostic factor, corresponding to the hazard ratio. The sum of all points results in a total score, from which a straight line can be drawn on a scale that displays the 2 and 5-year risk of readmission or reoperation. Statistics were performed in R (version 3.5.1) using the Regression Modelling Strategies Package [11]. 

To quantify the accuracy of the predictive discrimination of the constructed prediction model, the concordance statistic (c-statistic) was used [12]. The c-statistic is a measure of goodness of fit for binary outcomes in a logistic regression model. A c-statistic of 0.65–0.70 was considered moderate predictive discrimination, 0.70–0.79 was considered good predictive discrimination, 0.80–0.89 excellent, and >0.9 outstanding [13]. When a prediction model is constructed based on a single cohort, predictive discrimination may be overestimated and would be expected to perform less adequately on a random sample. To validate the constructed prediction model, internal cross-validation through a bootstrap procedure was performed [14]. Fifty random samples were created for the bootstrap resampling. On the bootstrap resampling procedure an adjusted c-statistic is calculated. The difference in the compared c-statistics is described as the optimism [14]. An optimism below 0.01 means an accurate model and retention of the original model.

## 3. Results

**Figure 1:**
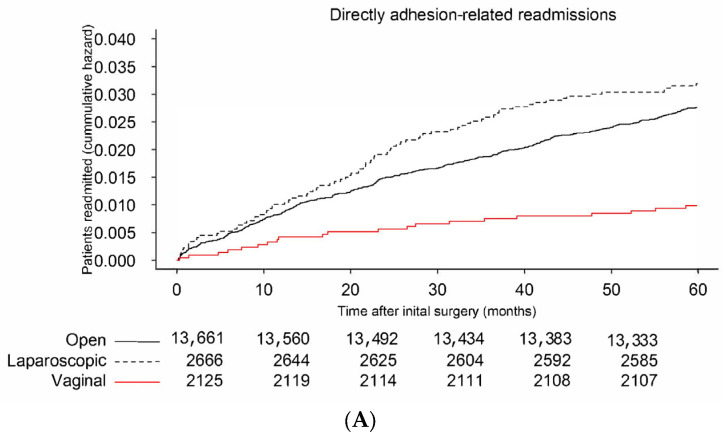

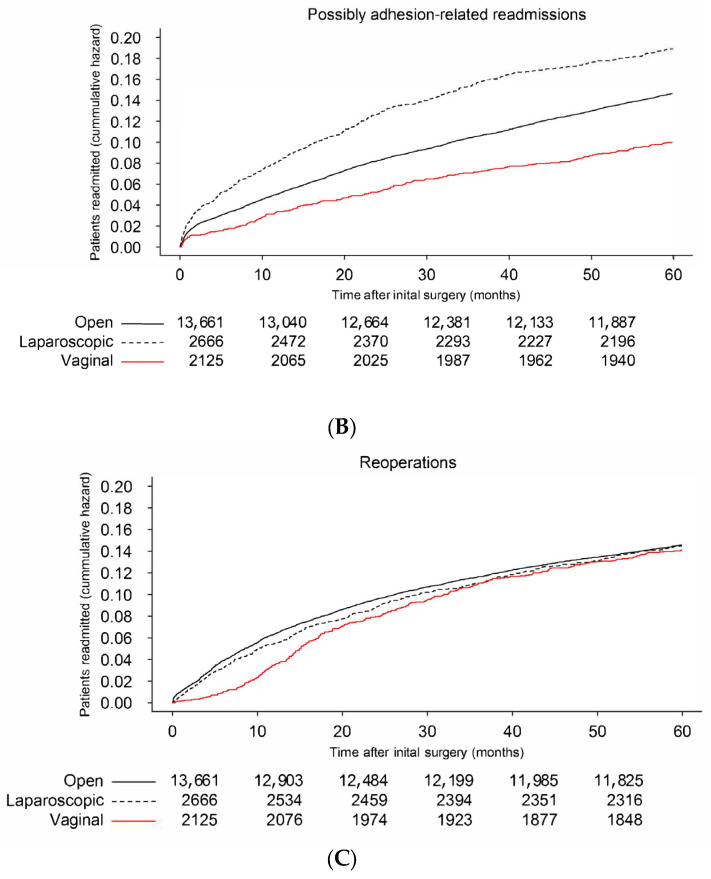
Kaplan–Meijer curve of directly adhesion-related readmissions (**A**), possibly adhesion-related readmissions (**B**), and reoperations after gynecological surgery (**C**).

During the two-year period, 18,452 women underwent their first gynecological surgery, by laparotomy in 13,661 (74.1%), by laparoscopy in 2666 (14.4%), and transvaginally in 2125 (11.5%) (Table 1). Over the five-year follow-up, 484 (2.6%) women were readmitted for a direct adhesion-related complication (Figure 1A) and 2719 (14.7%) women were readmitted for a complication possibly related to adhesions (Figure 1B). Reoperations were performed in 2679 (14.5%) women (Figure 1C). Hysterectomies as initial surgery were performed open in 69.6%, laparoscopically in 3.9%, and transvaginally in 26.5% (Table S1).

Baseline characteristics showed a small difference between the three surgical approaches in age, operation site, malignancy, fertility-enhancing surgery, mesh placement, endometriosis as diagnosis, and adhesiolysis at initial surgery (Table 2). Malignancy was the indication for surgery in 10.8%, endometriosis in 7.6%, and 4.6% of surgeries were performed to enhance fertility. 

It was not fully clear for all reoperations if these were related or unrelated to the previous surgery based on the available data. However, a large proportion of the operations seemed not to be related to the initial procedure. At least one in four open reoperations were not associated with initial procedure and one in three laparoscopic reoperations were not related to the initial procedure.

Baseline characteristics of women who underwent hysterectomy as initial surgery are shown in Supplementary Table S1. 

### 3.1. Risk Factors for Readmission Directly Related to Adhesions

**Figure 2:**
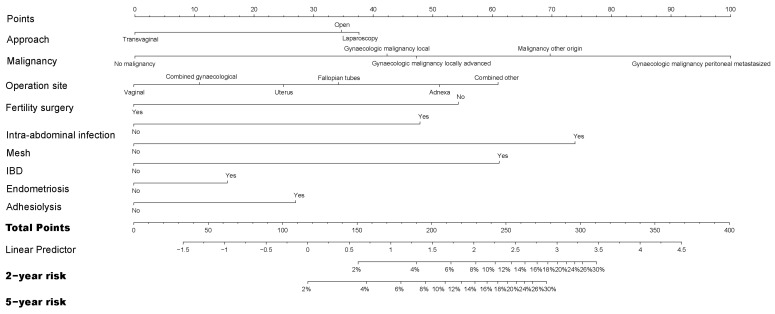
Nomogram to predict readmission directly related to adhesions in women who underwent gynecological surgery. By drawing a vertical line from each variable to the points axis on top and summing the individual points for all variables, the total score is calculated. From the total score axis perpendicular to the bottom, the linear predictor and the 2- and 5-year risk for reoperation are determined. IBD = inflammatory bowel disease.

The results of the univariable analysis are found in the Supplementary Table S2. In multivariable analysis (Table S3) gynecological malignancy with peritoneal metastasis (HR 5.95 95% CI 3.55–9.97) had the highest impact on the risk of directly related readmissions. Other risk factors included mesh placement (HR 3.77 95% CI 2.55–5.58), and endometriosis as indication for initial surgery (HR 1.32 95% CI 0.97–1.80). A nomogram was constructed to predict the risk of readmission directly related to adhesions after gynecological surgery, using a score based on the Hazard ratios (Figure 2). C-statistics of the original model and after cross-validation were comparable (0.711 and 0.703), with low optimism (0.0087). Therefore, the original model was retained. 

Univariable analysis for the risk of readmission directly related to adhesions in women after initial hysterectomy (Table S4) showed comparable risk factors to gynecological surgery in general. Noticeable is that IBD was also not a significant risk factor, and was not taken into the multivariable analysis (Table S5). The prediction model (nomogram in Supplementary Figure S1) had good predictive reliability (c-statistics 0.703 and 0.696). The low optimism (0.007) did not require adaptation of the original model.

### 3.2. Risk Factors for Possibly Adhesion-Related Readmissions

**Figure 3:**
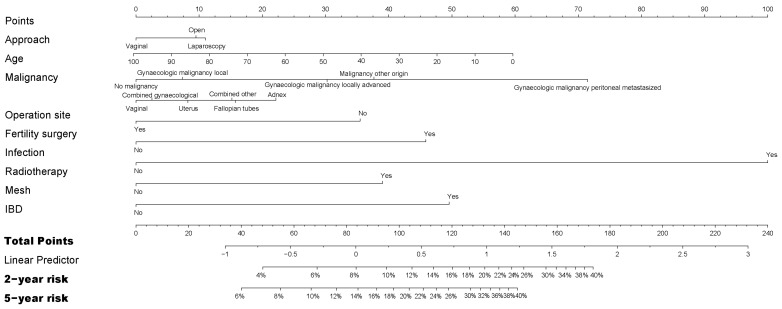
Nomogram to predict readmission possibly related to adhesions in women who underwent gynecological surgery. IBD = inflammatory bowel disease.

Risk factors for possibly adhesion-related readmission were largely comparable to the model for directly related readmission. Remarkably, endometriosis as indication for initial surgery did not significantly impact the risk in univariable analysis (Supplementary Table S6). In multivariable analysis (Table S7), both laparoscopic surgery (HR 1.25 95% CI 1.04–1.50) and open surgery (HR 1.21 95% CI 1.04–1.42) had an increased risk of readmission compared to transvaginal surgery. A nomogram was constructed for the prediction of possibly adhesion-related readmissions after gynecological surgery (Figure 3).

Comparable c-statistics were found for the original model and after internal cross-validation (0.611 and 0.609). Optimism in the original model (0.004) was low and the original prediction model was retained.

Comparable risk factors were identified for hysterectomies and gynecological surgery in general. Remarkably, in univariable analysis, IBD and adhesiolysis were not significant risk factors (Table S8). In multivariable analysis (Table S9), history of radiotherapy had the largest impact on the risk of readmission (HR 9.78 95% CI 4.85–19.72). The prediction model, displayed as a nomogram in Figure S2, had moderate predictive reliability (c-statistics 0.601 and 6.00). The original prediction model was retained due to low optimism (0.001). 

### 3.3. Risk Factors for Reoperation after Gynecological Surgery

**Figure 4:**
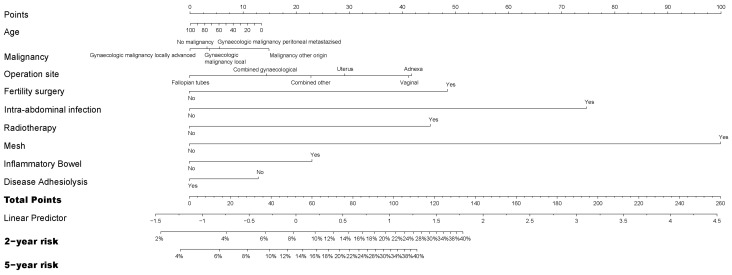
Nomogram to predict reoperation after initial gynecological surgery. IBD = inflammatory bowel disease.

The model of risk factors for reoperation had some differences compared to the models for readmissions. Overall, the surgical approach and endometriosis were not significant risk factors for reoperation in univariable analysis (Supplementary Table S10) and were not incorporated in the multivariable analysis (Table S11). Intra-abdominal infection and mesh placement had the highest impact on the risk for reoperation. The prediction model (Figure 4) showed comparable c-statistics to the original model after cross-validation (0.651 and 0.647). Owing to low optimism (0.0036), the original model was retained. 

Risk factors for reoperation after initial hysterectomy were comparable to general gynecology, except that surgical approach was significant, and operation site was not significant in univariable analysis for initial hysterectomies (Table S12). In multivariable analysis (Table S13) the risk of reoperation was highest in transvaginal performed procedures (14.1% HR 2.20 95% CI 1.44–3.35), followed by open (8.7% HR 1.06 95% CI 0.67–1.59) and laparoscopic procedures (8.0%). 

The nomogram is presented in the appendix (Figure S3). Internal validation of the prediction model showed comparable c-statistics (0.575 and 0.571). Low optimism (0.003) meant that adaptation of the original model was not required.

## 4. Discussion

### 4.1. Main Findings

Using the Scottish registry data, we established the risk factors for clinically relevant long-term adhesion-related consequences. Based on this nationwide cohort, nomograms were constructed to provide an evidence-based prediction model for the risk of readmission related to adhesions and reoperations in the individual woman undergoing initial gynecological surgery. The predictive value was moderate to good.

The risk of readmission for adhesion-related complications was 10-fold higher after procedures with hysterectomy compared to uterus-sparing surgeries, and more than 6-fold higher when women received prior radiotherapy compared to no history of radiotherapy. Reoperations were most frequent following hysterectomy and operations with mesh placement. Following transvaginal surgery one in ten women was readmitted for adhesion-related complications, compared to almost one in five following laparotomy or laparoscopy. Noticeable is the low impact on adhesion-related readmissions and reoperations found for the factors endometriosis and the need for adhesiolysis during the initial surgery. 

### 4.2. Strengths and Limitations 

In this study, we were able to quantify the impact of potential risk factors on clinically relevant outcomes of adhesion-related complications. Risk factors for adhesion-related complications were previously suggested based on clinical experience, without clinical validation [7]. Most suggested risk factors were confirmed, although some were not found to correlate with adhesion-related readmissions or reoperations.

The data are of high quality and representative for high-income countries. This database comprises extensive nationwide data of a population with a low level of migration, which enables analysis of real-world data.

A limitation of this study is the difficulty in accurately estimating the total disease burden of adhesions based on analysis of admission and operation codes. Complications such as infertility and chronic pelvic pain that usually do not require readmission are therefore often not analyzed. Another limitation is the difficulty in defining adhesion-related complications. Surgical exploration is needed for confirmation of adhesions as the primary cause of the symptoms, but most patients are treated non-operatively [15]. Readmissions were therefore coded as possibly related to adhesions. Analysis of directly related readmissions grossly underestimated the total burden of adhesions. When including possibly related readmissions, some readmissions that are not truly attributable to adhesions might also be included. In our analysis, risk factors for only the directly related readmissions and all possibly related readmissions were comparable, indicating that these are true risk factors for adhesion-related complications. 

Adhesion formation is effected by the extent of peritoneal injury, which is impacted by surgical technique and experience of the surgical team [7]. The nationwide database used in this study, however, was not granular enough to score items related to surgical expertise perioperative techniques (such as hemostasis, lavage, and use of anti-adhesion barriers). 

Mesh placement in our cohort of initial surgeries was most often used in the laparotomy group, and in particular used for reconstructions after extensive oncological resections or endometriosis resections. In daily gynecological practice, meshes are more frequently placed in reoperations and located in the lesser pelvis, as part of surgery for pelvic organ prolapse [16,17]. Organ prolapse surgery is often performed at a higher age and seldom as an initial operation. Nevertheless, mesh remained an independent risk factor after correcting for both malignancy and age.

Our constructed prediction model could not be validated on an external population. However, internal cross-validation using bootstrapping methods was applied. Internal cross-validation showed comparable c statistics and very low optimism for all prediction models, indicating reliable prediction models. 

### 4.3. Interpretation

Compared with the original SCAR cohort from two decades ago, the overall readmission rate after laparoscopic and open gynecological surgery remains largely the same [8]. The SCAR studies introduced the use of readmissions and reoperation as an outcome measuring long-term morbidity of adhesions. Most studies on long-term outcomes of adhesions focus on only one single complication, e.g., small bowel obstruction [18,19]. After open gynecological surgery, small bowel obstruction is reported in 7–18% of operations, which is comparable to the 3–14% of adhesions-related readmissions in this study. Following transvaginal hysterectomy, the reported small bowel obstruction rate is 2%, compared to 1–10% of adhesion-related readmissions in this study [19,20]. 

The results of our study show that the burden of adhesion following gynecological surgery remains very high. A risk that is often underestimated in clinical practice, as shown by a knowledge test in an adhesion survey among gynecologists [21]. Furthermore, only 40% of gynecologists inform patients about the risk of adhesions routinely for some surgeries, and only 20% inform patients routinely for all surgeries [22].

A remarkable finding is the high incidence of adhesion-related readmission in the laparoscopic group. Moreover, only 15% of procedures was performed laparoscopically, which is low compared to other studies, and even the original SCAR studies [8,23,24]. However, the original SCAR study also included diagnostic laparoscopies in which no further gynecological procedure was performed. The high number of adhesion-related readmissions after laparoscopy might partially be attributed to the case-mix and are not fully covered by OPCS-4 and ICD-10 codes, contributing to this result. Furthermore, a relatively large number of minimally invasive procedures were performed transvaginally, indicating that the simpler cases may have been performed transvaginal leaving the larger minimal invasive resections for laparoscopic surgery. Previous studies in other fields of surgery have demonstrated a lower risk of adhesion formation after laparoscopy as compared to open surgery. Nevertheless, colorectal studies have demonstrated that up to 48% of patients undergoing laparoscopic surgery, develop adhesions in the area of dissection [25], indicating that adhesions can still be problematic in case of larger laparoscopic dissection. 

In this study, endometriosis only impacted the risk for directly adhesion-related readmission. Endometriosis as indication for initial surgery was not a significant risk factor for reoperation and readmission possibly related to adhesions after gynecological surgery. This might be explained by the low number of reported endometrioses in ICD-10 codes. In recent literature, endometriosis is proposed as a factor for indirectly increasing adhesion formation after surgery [26]. Perturbed endocrine pathways, inflammatory responses, and tissue remodeling even increase the risk for adhesion formation without surgery [27].

Previous studies regarding risk factors for adhesion formation often assess intraoperative risks [19,28]. Our study addresses an important knowledge gap in preoperatively predicting the risk of readmission or reoperation after gynecological surgery. A few of the variables contributing to higher readmission or reoperation risk were confirmed in previous studies and minimally invasive hysterectomy resulted in less adhesion-related complications. Moreover, malignancy posed a significant risk factor for adhesion-related complications [18]. A previous study showed that radiotherapy is a risk factor for adhesion formation, although other possible confounding factors were not taken into consideration in that study [29].

The constructed prediction models can be used for better preoperative counseling of patients and can aid the process of shared decision-making. A tailored risk analysis of the individual risk of adhesion-related readmissions can aid in assessing the benefits and risks of an operation. The model could also guide the surgeon in the use of anti-adhesion barriers when a pre-operative high risk of adhesion-related complications or reoperations is calculated. In future studies the model might further be refined with peri- and intra-operative factors to guide the use of anti-adhesion barriers. Typically, barriers are installed at the end of the surgical procedure, which would allow reassessment of their need at the end of surgery with consideration of the operative course. Cut-off values for the cost-effective use of anti-adhesion barriers still need to be defined [30]. 

## 5. Conclusions

This national cohort study showed that one in seven women will be readmitted within five years after gynecological surgery and one in seven women will be reoperated on. The nomograms constructed in this study provide a prediction model of the two- and five-year risk for readmission or reoperation. Risk factors with the largest impact are a history of radiotherapy and mesh placement. 

## Figures and Tables

**Table 1:** Number of readmissions and reoperations.

	Laparotomy N = 13,661	Laparoscopy N = 2666	Transvaginal N = 2125	Total N = 18,452
Number women of readmitted	2003 (14.6%)	2003 (14.6%)	212 (10.0%)	2719 (14.7%)
Ovary	170 (14.3%)	170 (14.3%)	-	
Fallopian tubes	128 (20.9%)	128 (20.9%)	-	
Vagina	388 (11.0%)	388 (11.0%)	-	
Uterus	318 (14.2%)	318 (14.2%)	72 (17.8%)	
Combined gynecological	518 (14.2%)	518 (14.2%)	133 (8.4%)	
Other combined	482 (19.6%)	482 (19.6%)	7 (5.4%)	
Number of total readmissions	3033	896	274	4203
Ovary	258	504	-	
Fallopian tubes	204	121	-	
Vagina	498	-	-	
Uterus	510	37	102	
Combined gynecological	797	111	163	
Other combined	766	123	9	
Number of women undergoing reoperation	1993 (14.6%)	386 (11.3%)	300 (14.1%)	2679 (14.5%)
Ovary	377 (31.8%)	205 (18.8%)	-	
Fallopian tubes	55 (9.0%)	27 (6.6%)	-	
Vagina	611 (17.3%)	-	-	
Uterus	316 (14.1%)	19 (11.1%)	59 (14.6%)	
Combined gynecological	317 (8.7%)	81 (11.6%)	221 (13.9%)	
Other combined	317 (12.9%)	54 (17.8%)	20 (15.4%)	

**Table 2:** Baseline characteristics.

	Laparotomy	Laparoscopy	Transvaginal	Total	Sig.
Age (years)					*p* < 0.001
Min	0	0	25	0	
Max	94	99	89	99	
Mean (SD)	51.27 (14.69)	39.17 (14.44)	59.38 (12.10)	50.46 (15.31)	
Operation site					*p* < 0.001
Ovary	1186 (8.7%)	1088 (40.8%)	0	2274 (12.3%)	
Fallopian tubes	611 (4.5%)	407 (15.3%)	0	1018 (5.5%)	
Vagina	3523 (25.8%)	0	0	3523 (19.1%)	
Uterus	316 (14.1%)	171 (6.4%)	404 (19.0%)	2815 (15.3%)	
Combined gynecological	317 (8.7%)	696 (26.1%)	1591 (74.9%)	5928 (32.1%)	
Combined other	317 (12.9%)	304 (11.4%)	130 (6.1%)	2894 (15.7%)	
Malignancy					*p* < 0.001
No malignancy	11,923 (87.3%)	2453 (92.0%)	2079 (97.8%)	16,455 (89.2%)	
Gynecological malignancy local	853 (6.2%)	115 (4.3%)	32 (1.5%)	1000 (5.4%)	
Gyn malignancy locally advanced	547 (4.0%)	32 (1.2%)	6 (0.3%)	585 (3.2%)	
Gyn malignancy peritoneal metastasized	71 (0.5%)	10 (0.4%)	0	81 (0.4%)	
Malignancy other origin	267 (2.0%)	56 (2.1%)	8 (0.4%)	331 (1.8%)	
Fertility enhancing surgery					*p* < 0.001
No	12,899 (94.4%)	2583 (96.9%)	2125 (100%)	17,607 (95.4%)	
Yes	762 (5.6%)	83 (3.1%)	0	845 (4.6%)	
Intra-abdominal infection					*p* = 0.214
No	13,603 (99.6%)	2653 (99.5%)	2121 (99.8%)	18,377 (99.6%)	
Yes	58 (0.4%)	13 (0.5%)	4 (0.2%)	75 (0.4%)	
History of radiotherapy					*p* = 0.309
No	13,645 (99.9%)	2664 (99.9%)	2125 (100%)	18,434 (99.9%)	
Yes	16 (0.1%)	2 (0.1%)	0	18 (0.1%)	
Mesh placement					*p* < 0.001
No	13,487 (98.7%)	2641 (99.1%)	2119 (99.7%)	18,247 (98.9%)	
Yes	174 (1.3%)	25 (0.9%)	6 (0.3%)	205 (1.1%)	
IBD					*p* = 0.285
No	13,603 (99.6%)	2657 (99.7%)	2121 (99.8%)	18,381 (99.6%)	
Yes	58 (0.4%)	9 (0.3%)	4 (0.2%)	71 (0.4%)	
Endometriosis					*p* = 0.004
No	12,747 (93.3%)	2302 (86.3%)	1999 (94.1%)	17,048 (92.4%)	
Yes	914 (6.7%)	364 (13.7%)	126 (5.9%)	1404 (7.6%)	
Adhesiolysis					*p* < 0.001
No	13,276 (97.%)	2649 (99.4%)	2122 (99.9%)	18,047 (97.8%)	
Yes	385 (2.8%)	17 (0.6%)	3 (0.1%)	204 (2.2%)	

## Data Availability

Data subject to third party restrictions. The data that support the findings of this study are available from the Scottish National Health Service (NHS). Restrictions apply to the availability of these data, which were used under license for this study. Data derived from the analysis are available from the authors with the permission of the Scottish NHS. The used repository was the Safe Haven (https://shs.epcc.ed.ac.uk/2fa/scotnsh.html, accessed 19 August 2019) from the NHS.

## Supplementary Materials

The following supporting information can be downloaded at: https://www.mdpi.com/article/10.3390/jcm12041351/s1, Table S1: Baseline characteristics of women undergoing baseline hysterectomy. Table S2: Univariate analysis of readmission directly related to adhesions in women who underwent index gynecological surgery. Table S3: Multivariate analysis of readmission directly related to adhesions in women who underwent initial gynecological surgery. Table S4: Univariate analysis of readmission directly related to adhesions in women who underwent initial hysterectomy. Table S5: Multivariate analysis of readmission directly related to adhesions in women who underwent initial hysterectomy. Table S6: Univariate analysis of risk for readmissions directly or possibly related to adhesions in women after gynecological surgery. Table S7: Multivariate analysis of risk for readmissions directly or possibly related to adhesions in women after gynecological surgery. Table S8: Univariate analysis of risk for readmission directly or possibly related to adhesions in women undergoing initial hysterectomy. Table S9: Multivariate analysis of risk for readmission directly or possibly related to adhesions in women undergoing initial hysterectomy. Table S10: Univariate analysis of reoperation in women who underwent initial gynecological surgery. Table S11: Multivariate analysis of reoperation in women who underwent initial gynecological surgery. Table S12: Univariate analysis of reoperation in women who underwent initial hysterectomy. Table S13: Multivariate analysis of reoperation in women who underwent initial hysterectomy. Figure S1: Nomogram to predict readmission directly related to adhesions in woman who underwent initial hysterectomy. Figure S2: Nomogram to predict readmission possibly related to adhesions in woman who underwent initial hysterectomy. Figure S3: Nomogram to predict reoperation in woman who underwent initial hysterectomy.
