# Supplementary material for: Risk Factors for Adhesion-Related Readmission and Abdominal Reoperation after Gynecological Surgery: A Nationwide Cohort Study
Source: J Clin Med. 2023 Feb 8;12(4):1351. doi: 10.3390/jcm12041351 (PMC9965311; doi:10.3390/jcm12041351)

Supplements

Risk Factors for Adhesion-Related Readmission and Abdominal Reoperation after Gynecological Surgery: A Nationwide Cohort Study

Masja Toneman [1,*], Tjitske Groenveld [1], Pepijn Krielen [1], Angelo Hooker [2], Rudy de Wilde [3], Luz Angela Torres-de la Roche [3], Atillio Di Spiezio Sardo [4], Philippe Koninckx [5], Ying Cheong [6,7], Annemiek Nap [8], Harry van Goor [1], Pille Pargmae [8] and Richard ten Broek [1]

1 Department of Surgery, Radboudumc, 6525 GA Nijmegen, The Netherlands
2 Department of Obstetrics and Gynecology, Zaans Medical Center (ZMC), 1502 DV Zaandam, The Netherlands
3 University Hospital for Gynecology, Carl von Ossietzky University, 26121 Oldenburg, Germany
4 Department of Public Health, School of Medicine, University of Naples Federico II, 80131 Naples, Italy
5 Department of Gynecology, Katholieke Universiteit Leuven, 3000 Leuven, Belgium
6 Faculty of Medicine, University of Southampton, Southampton SO16 6YD, UK
7 Complete Fertility Centre, Southampton SO16 5YA, UK
8 Department of Gynecology, Radboudumc, 6525 GA Nijmegen, The Netherlands
* Correspondence: masja.toneman@radboudumc.nl

Table S1. Baseline characteristics of woman undergoing baseline hysterectomy.

	**Open**	**Laparoscopy**	**Transvaginal**	**Total**	**Sig**
Patients	5594	313	2125	8032	
Age					P<0.001
Min	15	14	25	14	
Max	93	89	89	93	
Mean	52.2 (12.04)	51.55 (13.28)	59.4 (12.10)	54.07 (12.52)	
Operation level					P<0.001
Uterus	1149 (20.5%)	120 (38.3%)	404 (19.0%)	1673 (20.8%)	
Combined gynecological	2969 (53.1%)	148 (47.3%)	1591 (74.9%)	4708 (58.6%)	
Combined other	1476 (26.4%)	45 (14.4%)	130 (6.1%)	1651 (20.6%)	
Malignancy					P<0.001
No malignancy	4154 (74.3%)	202 (64.5%)	2079 (97.8%)	6435 (80.1%)	
Gynecological malignancy local	778 (13.9%)	89 (28.4%)	32 (1.5%)	899 (11.2%)	
Gyn. malignancy locally advanced	477 (8.5%)	18 (5.8%)	6 (0.3%)	501 (6.2%)	
Gyn. malignancy peritoneal metastasized	52 (0.9%)	0	0	52 (0.6%)	
Malignancy other origin	133 (2.4%)	4 (1.3%)	8 (0.4%)	145 (1.8%)	
Intra-abdominal infection					P=0.135
No	5569 (99.6%)	313 (100%)	2121 (99.8%)	8003 (99.6%)	
Yes	25 (0.4%)	0	4 (0.2%)	29 (0.4%)	
History of radiotherapy					P=0.010
No	5585 (99.8%)	311 (99.4%)	2125 (100%)	8021 (99.9%)	
Yes	9 (0.2%)	2 (0.6%)	0	11 (0.1%)	
Mesh placement					P<0.001
No	5495 (98.2%)	309 (98.7%)	2119 (99.7%)	7923 (98.6%)	
Yes	99 (1.8%)	4 (1.3%)	6 (0.3%)	109 (1.4%)	
IBD					P=0.882
No	5582 (99.8%)	312 (99.7%)	2121 (99.8%)	8015 (99.8%)	
Yes	12 (0.2%)	1 (0.3%)	4 (0.2%)	17 (0.2%)	
Endometriosis					P<0.001
No	4887 (87.4%)	285 (91.1%)	1999 (94.1%)	7171 (89.3%)	
Yes	707 (12.6%)	28 (8.9%)	126 (5.9%)	861 (10.7%)	
Adhesiolysis					P<0.001
No	5319 (94.9%)	300 (98.4%)	2122 (99.9%)	7741 (96.4%)	
Yes	283 (5.1%)	5 (1.6%)	3 (0.1%)	291 (3.6%)	

Table S2: Univariate analysis of readmission directly related to adhesions in woman who underwent index gynecological surgery

	N/N total (%)	HR (95% CI)	Sig.
Approach			
Open	378/13661 (2.8%)	2.824 (1.820-4.382)	p<0.001
Laparoscopic	85/2666 (3.2%)	3.266 (2.026-5.266)	
Vaginal	21/2125 (1.0%)	Ref.	
Malignant disease			p<0.001
No malignancy	334/16455 (2.0%)	Ref.	
Gynecological malignancy local	38/1000 (3.8%)	1.890 (1.351-2.644)	
Gynecological malignancy locally advanced	57/585 (9.7%)	5.029 (3.798-6.660)	
Gynecological malignancy peritoneal metastasized	17/81 (21.0%)	11.576 (7.110-18.845)	
Malignancy other origin	38/331 (11.5%)	6.021 (4.304-8.421)	
Operation site			p<0.001
Ovary	65/2274 (2.9%)	2.261 (1.546-3.306)	
Fallopian Tubes	24/1018 (2.4%)	1.854 (1.130-3.043)	
Vagina	45/3523 (1.3%)	Ref.	
Uterus	57/2815 (2.0%)	1.594 (1.078-2.356)	
Combined gynecologic	105/5928 (1.8%)	1.394 (0.983-1.976)	
Combined other	188/2894 (6.5%)	5.255 (3.796-7.276)	
Fertility surgery			
No	474/17607 (2.7%)	Ref.	
Yes	10/845 (1.2%)	0.436 (0.233-0.815)	P=0.009
Intra-abdominal infection			
No	478/18377 (2.6%)	Ref.	
Yes	6/75 (8.0%)	3.135 (1.401-7.012)	P=0.005
History of radiotherapy			
No	483/18434 (2.6%)	Ref.	
Yes	1/18 (5.6%)	1.934 (0.648-3.471)	P=0.486
Mesh placement			
No	456/18247 (2.5%)	Ref.	
Yes	28/205 (13.7%)	5.761 (3.933-8.438)	p<0.001
IBD			
No	476/18381 (2.6%)	Ref.	
Yes	8/71 (11.3%)	4.584 (2.279-9.220)	p<0.001
Endometriosis			
No	436/17048 (2.6%)	Ref.	
Yes	48/1404 (3.4%)	1.341 (0.996-1.807)	P=0.053
Adhesiolysis			
No	450/18047 (2.5%)	Ref.	
Yes	34/405 (8.4%)	3.484 (2.458-4.937)	p<0.001

Table S3: multivariate analysis of readmission directly related to adhesions in woman who underwent initial gynecological surgery

	**N/N total (%)**	**HR (95% CI)**	**Sig.**
Approach			p=0.029
Open	378/13661 (2.8%)	1.854 (1.164-2.954)	
Laparoscopic	85/2666 (3.2%)	1.952 (1.153-3.303)	
Transvaginal	21/2125 (1.0%)	Ref.	
Age		1.000 (0.992-1.007)	p=0.910
Malignant disease			p<0.001
No malignancy	334/16455 (2.0%)	Ref.	
Gynecological malignancy local	38/1000 (3.8%)	2.144 (1.462-3.146)	
Gynecological malignancy locally advanced	57/585 (9.7%)	2.340 (1.662-3.296)	
Gynecological malignancy peritoneal metastasized	17/81 (21.0%)	5.950 (3.552-9.968)	
Malignancy other origin	38/331 (11.5%)	3.504 (2.410-5.095)	
Operation site			p<0.001
Ovary	65/2274 (2.9%)	2.488 (1.569-3.944)	
Fallopian Tubes	24/1018 (2.4%)	1.830 (1.063-3.151)	
Vagina	45/3523 (1.3%)	Ref.	
Uterus	57/2815 (2.0%)	1.559 (1.033-2.354)	
Combined gynecologic	105/5928 (1.8%)	1.214 (0.825-1.787)	
Combined other	188/2894 (6.5%)	2.984 (2.063-4.315)	
Fertility enhancing surgery			
No	474/17607 (2.7%)	2.663 (1.344-5.277)	
Yes	10/845 (1.2%)	Ref.	P=0.005
Intra-abdominal infection			
No	478/18377 (2.6%)	Ref.	
Yes	6/75 (8.0%)	2.363 (1.046-5.338)	P=0.039
Mesh placement			
No	456/18247 (2.5%)	Ref.	
Yes	28/205 (13.7%)	3.772 (2.551-5.577)	p<0.001
IBD			
No	476/18381 (2.6%)	Ref.	
Yes	8/71 (11.3%)	2.998 (1.472-6.105)	P=0.002
Endometriosis			
No	436/17048 (2.6%)	Ref.	
Yes	48/1404 (3.4%)	1.323 (0.970-1.803)	P=0.077
Adhesiolysis			
No	450/18047 (2.5%)	Ref.	
Yes	34/405 (8.4%)	1.629 (1.130-2.347)	P=0.009

Table S4: Univariate analysis of readmission directly related to adhesions in woman who underwent initial hysterectomy.

	N/N total (%)	HR (95% CI)	Sig.
Approach			
Open	189/5594 (3.4%)	3.459 (2.204-5.430)	P<0.001
Laparoscopic	10/313 (3.2%)	3.286 (1.548-6.978)	
Vaginal	21/2125 (1.0%)	Ref.	
Age			
Operation site			
Uterus	35/1673 (2.1%)	1.188 (0.800-1.763)	P<0.001
Combined gynecologic	83/4708 (1.8%)	Ref.	
Combined other	102/1651 (6.2%)	3.600 (2.695-4.860)	
Malignant disease			
No malignancy	114/6435 (1.8%)	Ref.	P<0.001
Gynecological malignancy local	32/899 (3.6%)	2.025 (1.368-2.997)	
Gynecological malignancy locally advanced	48/501 (9.6%)	5.639 (4.025-7.901)	
Gynecological malignancy peritoneal metastasized	8/52 (15.4%)	9.229 (4.506-18.901)	
Malignancy other origin	18/145 (12.4%)	7.511 (4.569-12.348)	
Intra-abdominal infection			
No	217/8003 (2.7%)	Ref.	
Yes	3/29 (10.3%)	3.904 (1.249-12.199)	P=0.019
History of radiotherapy			
No	219/8021 (2.7%)	Ref.	
Yes	1/11 (9.1%)	3.566 (0.500-25.431)	P=0.205
Mesh placement			
No	205/7923 (2.6%)	Ref.	
Yes	15/109 (13.8%)	5.595 (3.312 -9.452)	P<0.001
IBD			
No	220/8015 (2.7%)	Ref.	
Yes	0/17 (0.0%)	0.050 (0.000-17840.829)	P=0.645
Endometriosis			
No	205/7171 (2.9%)	Ref.	
Yes	15/861 (1.7%)	0.605 (0.358-1.022)	P=0.060
Adhesiolysis			
No	202/7741 (2.6%)	Ref.	
Yes	18/291 (6.2%)	2.405 (1.485-3.894)	P<0.001

Table S5: multivariate analysis of readmission directly related to adhesions in woman who underwent initial hysterectomy.

	N/N total (%)	HR (95% CI)	Sig.
Approach			P=0.017
Open	189/5594 (3.4%)	2.043 (1.249-3.343)	
Laparoscopic	10/313 (3.2%)	2.109 (0.961-4.628)	
Transvaginal	21/2125 (1.0%)	Ref.	
Age		1.002 (0.990-1.015)	P=0.711
Malignant disease			P<0.001
No malignancy	114/6435 (1.8%)	Ref.	
Gynecological malignancy local	32/899 (3.6%)	1.885 (1.201-2.958)	
Gynecological malignancy locally advanced	48/501 (9.6%)	2.809 (1.775-4.445)	
Gynecological malignancy peritoneal metastasized	8/52 (15.4%)	5.005 (2.313-10.829)	
Malignancy other origin	18/145 (12.4%)	4.248 (2.395-7.535)	
Operation site			P=0.025
Uterus	35/1673 (2.1%)	1.305 (0.0851-2.001)	
Combined gynecological	83/4708 (1.8%)	Ref.	
Combined other	102/1651 (6.2%)	1.805 (1.171-2.780)	
Intra-abdominal infection			
No	217/8003 (2.7%)	Ref.	
Yes	3/29 (10.3%)	2.324 (0.716-7.548)	P=0.160
Mesh placement			
No	205/7923 (2.6%)	Ref.	
Yes	15/109 (13.8%)	3.665 (2.126-6.317)	P<0.001
Endometriosis			
No	205/7171 (2.9%)	Ref.	
Yes	15/861 (1.7%)	0.731 (0.427-1.251)	P=0.253
Adhesiolysis			
No	202/7741 (2.6%)	Ref.	
Yes	18/291 (6.2%)	1.201 (0.729-1.978)	P=0.472

Table S6: Univariate analysis of risk for readmissions possibly related to adhesions in woman after gynecological surgery.

	N/N total (%)	HR (95% CI)	Sig.
Approach			
Open	2003/13661 (14.7%)	1.510 (1.310-1.739)	p<0.001
Laparoscopic	504/2666 (18.9%)	2.022 (1.722-2.374)	
Vaginal	212/2125 (10.0%)	Ref.	
Malignant disease			
No malignancy	2303/16455 (14.0%)	Ref.	P<0.001
Gynecological malignancy local	141/1000 (14.1%)	1.006 (0.848-1.192)	
Gynecological malignancy locally advanced	148/585 (25.3%)	1.961 (1.660-2.315)	
Gynecological malignancy peritoneal metastasized	38/81 (46.9%)	4.316 (3.132-5.948)	
Malignancy other origin	89/331 (26.9%)	2.128 (1.722-2.630)	
Operation site			
Ovary	420/2274 (18.5%)	1.621 (1.372-1.914)	P<0.001
Fallopian Tubes	202/1018 (19.8%)	1.445 (1.187-1.761)	
Vagina	388/3523 (11.0%)	Ref.	
Uterus	413/2815 (14.7%)	1.190 (1.025-1.381)	
Combined gynecologic	729/5928 (12.3%)	1.074 (0.937-1.231)	
Combined other	567/2894 (19.6%)	1.375 (1.186-1.595)	
Fertility surgery			
No	2626/17607 (14.9%)	Ref.	
Yes	93/845 (11.0%)	0.728 (0.592-0.896)	p=0.003
Intra-abdominal infection			
No	2692/18377 (14.6%)	Ref.	
Yes	27/75 (36.0%)	2.538 (1.734-3.716)	p<0.001
History of radiotherapy			
No	2707/18434 (14.7%)	Ref.	
Yes	12/18 (66.7%)	6.512 (3.671-11.552)	p<0.001
Mesh placement			
No	2650/18247 (14.5%)	Ref.	
Yes	69/205 (33.7%)	2.186 (1.717-2.783)	p<0.001
IBD			
No	2690/18381 (14.6%)	Ref.	
Yes	29/71 (40.8%)	2.716 (1.876-3.931)	p<0.001
Endometriosis			
No	2504/17048 (14.7%)	Ref.	
Yes	215/1404 (15.3%)	1.044 (0.908-1.200)	p=0.548
Adhesiolysis			
No	2648/18047 (14.7%)	Ref.	
Yes	71/405 (17.5%)	1.224 (0.967-1.549)	P=0.093

Table S7: Multivariate analysis of risk for readmissions possibly related to adhesions in woman after gynecological surgery.

	N/N total (%)	HR (95% CI)	Sig.
Approach			
Open	2003/13661 (14.7%)	1.212 (1.039-1.415)	P=0.009
Laparoscopic	504/2666 (18.9%)	1.249 (1.039-1.501)	
Transvaginal	212/2125 (10.0%)	Ref.	
Age		0.988 (0.985-0.991)	p<0.001
Malignant disease			
No malignancy	2303/16455 (14.0%)	Ref.	
Gynecological malignancy local	141/1000 (14.1%)	1.163 (0.967-1.399)	P<0.001
Gynecological malignancy locally advanced	148/585 (25.3%)	1.843 (1.516-2.242)	
Gynecological malignancy peritoneal metastasized	38/81 (46.9%)	4.228 (3.031-5.898)	
Malignancy other origin	89/331 (26.9%)	2.135 (1.708-2.670)	
Operation site			
Ovary	420/2274 (18.5%)	1.563 (1.307-1.870)	P<0.001
Fallopian Tubes	202/1018 (19.8%)	1.374 (1.129-1.671)	
Vagina	388/3523 (11.0%)	Ref.	
Uterus	413/2815 (14.7%)	1.180 (1.017-1.370)	
Combined gynecological	729/5928 (12.3%)	1.063 (0.919-1.208)	
Combined other	567/2894 (19.6%)	1.361 (1.172-1.580)	
Fertility enhancing surgery			
No	2626/17607 (14.9%)	2.045 (1.618-2.586)	
Yes	93/845 (11.0%)	Ref.	p<0.001
Intra-abdominal infection			
No	2692/18377 (14.6%)	Ref.	
Yes	27/75 (36.0%)	2.521 (1.721-3.690)	p<0.001
History of radiotherapy			
No	2707/18434 (14.7%)	Ref.	
Yes	12/18 (66.7%)	6.564 (3.699-11.646)	p<0.001
Mesh placement			
No	2650/18247 (14.5%)	Ref.	
Yes	69/205 (33.7%)	2.193 (1.723-2.793)	p<0.001
IBD			
No	2690/18381 (14.6%)	Ref.	
Yes	29/71 (40.8%)	2.716 (1.876-3.932)	p<0.001
Adhesiolysis			
No	2648/18047 (14.7%)	Ref.	
Yes	71/405 (17.5%)	0.964 (0.755-1.230)	p=0.768

Table S8: Univariate analysis of risk for readmission possibly related to adhesions in woman undergoing initial hysterectomy.

	N/N total (%)	HR (95% CI)	Sig.
Approach			
Open	837/5594 (15.0%)	1.545 (1.329-1.796)	P<0.001
Laparoscopic	42/313 (13.4%)	1.391 (0.999-1.937)	
Vaginal	212/2125 (10%)	Ref.	
Age			
Operation site			
Uterus	250/1673 (14.9%)	1.300 (1.120-1.510)	P<0.001
Combined gynecologic	552/4708 (11.7%)	Ref.	
Combined other	289/1651 (17.5%)	1.558 (1.351-1.796)	
Malignant disease			
No malignancy	793/6435 (12.3%)	Ref.	P<0.001
Gynecological malignancy local	116/899 (12.9%)	1.046 (0.861-1.271)	
Gynecological malignancy locally advanced	117/501 (23.4%)	2.047 (1.686-2.486)	
Gynecological malignancy peritoneal metastasized	22/52 (42.3%)	4.095 (2.681-6.255)	
Malignancy other origin	43/145 (29.7%)	2.755 (2.027-3.744)	
Intra-abdominal infection			
No	1078/8003 (13.5%)	Ref.	
Yes	13/29 (44.8%)	4.043 (2.340-6.987)	P<0.001
History of radiotherapy			
No	1083/8021 (13.5%)	Ref.	
Yes	8/11 (72.7%)	9.642 (4.807-19.342)	P<0.001
Mesh placement			
No	1060/7923 (13.4%)	Ref.	
Yes	31/109 (28.4%)	2.288 (1.601-3.270)	P<0.001
IBD			
No	1088/8015 (13.6%)	Ref.	
Yes	3/17 (17.6%)	1.391 (0.448-4.320)	P=0.568
Endometriosis			
No	980/7171 (13.7%)	Ref.	
Yes	111/861 (12.9%)	0.935 (0.768-1.137)	P=0.500
Adhesiolysis			
No	1045/7741 (13.5%)	Ref.	
Yes	46/291 (15.8%)	1.187 (0.884-1.595)	P=0.255

Table S9: Multivariate analysis of risk for readmission possibly related to adhesions in woman undergoing initial hysterectomy.

	N/N total (%)	HR (95% CI)	Sig.
Approach			
Open	837/5594 (15.0%)	1.183 (1.001-1.399)	P=0.129
Laparoscopic	42/313 (13.4%)	1.072 (0.760-1.511)	
Transvaginal	212/2125 (10%)	Ref.	
Age		0.987 (0.981-0.993)	P<0.001
Operation site			
Uterus	250/1673 (14.9%)	1.149 (0.916-1.441)	P=0.228
Combined gynecological	552/4708 (11.7%)	1.002 (0.822-1.220)	
Combined other	289/1651 (17.5%)	Ref.	
Malignant disease			
No malignancy	793/6435 (12.3%)	Ref.	P<0.001
Gynecological malignancy local	116/899 (12.9%)	1.112 (0.900-1.375)	
Gynecological malignancy locally advanced	117/501 (23.4%)	2.274 (1.761-2.937)	
Gynecological malignancy peritoneal metastasized	22/52 (42.3%)	4.577 (2.918-7.180)	
Malignancy other origin	43/145 (29.7%)	3.251 (2.327-4.541)	
Intra-abdominal infection			
No	1078/8003 (13.5%)	Ref.	
Yes	13/29 (44.8%)	3.712 (2.142-6.432)	P<0.001
History of radiotherapy			
No	1083/8021 (13.5%)	Ref.	
Yes	8.11 (72.7%)	9.776 (4.847-19.718)	P<0.001
Mesh placement			
No	1060/7923 (13.4%)	Ref.	
Yes	31/109 (28.4%)	1.898 (1.322-2.725)	P=0.001

Table S10: Univariate analysis of reoperation in woman who underwent initial gynecological surgery.

	**N/N total (%)**	**HR (95% CI)**	**Sig.**
Approach			P=0.713
Open	1993/13661 (14.6%)	1.052 (0.931-1.187)	
Laparoscopic	386/2666 (11.3%)	1.038 (0.892-1.207)	
Vaginal	300/2125 (14.1%)	Ref.	
Malignant disease			P=0.009
No malignancy	2436/16455 (14.8%)	Ref.	
Gynecological malignancy local	109/1000 (10.9%)	0.730 (0.602-0.884)	
Gynecological malignancy locally advanced	72/585 (12.3%)	0.815 (0.645-1.030)	
Gynecological malignancy peritoneal metastasized	10/81 (12.3%)	0.849 (0.456-1.581)	
Malignancy other origin	52/331 (15.7%)	1.084 (0.823-1.426)	
Operation site			p<0.001
Ovary	582/2274 (25.6%)	3.690 (2.928-4.650)	
Fallopian Tubes	82/1018 (8.1%)	Ref.	
Vagina	611/3523 (17.3%)	2.263 (1.797-2.850)	
Uterus	394/2815 (14.0%)	1.813 (1.429-2.300)	
Combined gynecologic	619/5928 (10.4%)	1.316 (1.045-1.657)	
Combined other	391/2894 (13.5%)	1.741 (1.372-2.209)	
Fertility surgery			
No	2355/17607 (13.4%)	Ref.	
Yes	324/845 (38.3%)	3.810 (3.392-4.279)	p<0.001
Intra-abdominal infection			
No	2624/18377 (14.3%)	Ref.	
Yes	55/75 (73.3%)	6.335 (4.849-8.275)	p<0.001
History of radiotherapy			
No	2674/18434 (14.5%)	Ref.	
Yes	5/18 (27.8%)	2.053 (0.854-4.935)	P=0.108
Mesh placement			
No	2521/18247 (13.8%)	Ref.	
Yes	158/205 (77.1%)	7.981 (6.792-9.377)	p<0.001
IBD			
No	2657/18381 (14.5%)	Ref.	
Yes	22/71 (31.0%)	2.293 (1.507-3.489)	p<0.001
Endometriosis			
No	2481/17048 (14.6%)	Ref.	
Yes	198/1404 (14.1%)	0.949 (0.821-1.096)	P=0.476
Adhesiolysis			
No	2641/18047 (14.6%)	Ref.	
Yes	38/405 (9.4%)	0.627 (0.455-0.863)	P=0.004

Table S11: multivariate analysis of reoperation in woman who underwent initial gynecological surgery.

	**N/N total (%)**	**HR (95% CI)**	**Sig.**
Approach			
Open	1993/13661 (14.6%)		
Laparoscopic	386/2666 (11.3%)		
Transvaginal	300/2125 (14.1%)		
Age		0.997 (0.994-1.000)	P=0.071
Malignant disease			
No malignancy	2436/16455 (14.8%)	Ref.	P=0.182
Gynecological malignancy local	109/1000 (10.9%)	0.991 (0.809-1.213)	
Gynecological malignancy locally advanced	72/585 (12.3%)	0.915 (0.705-1.186)	
Gynecological malignancy peritoneal metastasized	10/81 (12.3%)	1.035 (0.552-1.940)	
Malignancy other origin	52/331 (15.7%)	1.289 (0.968-1.718)	
Operation site			p<0.001
Ovary	582/2274 (25.6%)	2.496 (1.958-3.182)	
Fallopian Tubes	82/1018 (8.1%)	Ref.	
Vagina	611/3523 (17.3%)	2.474 (1.929-3.172)	
Uterus	394/2815 (14.0%)	1.901 (1.492-2.422)	
Combined gynecologic	619/5928 (10.4%)	1.374 (1.079-1.749)	
Combined other	391/2894 (13.5%)	1.629 (1.262-2.104)	
Fertility enhancing surgery			
No	2355/17607 (13.4%)	Ref.	
Yes	324/845 (38.3%)	2.889 (2.478-3.367)	p<0.001
Intra-abdominal infection			
No	2624/18377 (14.3%)	Ref.	
Yes	55/75 (73.3%)	5.089 (3.886-6.665)	p<0.001
History of radiotherapy			
No	2674/18434 (14.5%)	Ref.	
Yes	5/18 (27.8%)	2.684 (1.111-6.482)	P=0.028
Mesh placement			
No	2521/18247 (13.8%)	Ref.	
Yes	158/205 (77.1%)	8.848 (7.494-10.446)	p<0.001
IBD			
No	2657/18381 (14.5%)	Ref.	
Yes	22/71 (31.0%)	1.645 (1.077-2.512)	P=0.020
Adhesiolysis			
No	2641/18047 (14.6%)	Ref.	
Yes	38/405 (9.4%)	0.813 (0.621-1.067)	P=0.137

Table S12: Univariate analysis of reoperation in woman who underwent initial hysterectomy.

	N/N total (%)	HR (95% CI)	Sig.
Approach			
Open	487/5594 (8.7%)	1.098 (0.735-1.641)	P<0.001
Laparoscopic	25/313 (8.0%)	Ref.	
Vaginal	300/2125 (14.1%)	1.833 (1.219-2.757)	
Age			
Operation site			
Uterus	170/1673 (10.2%)	1.030 (0.864-1.227)	P=0.572
Combined gynecologic	465/4708 (9.9%)	Ref.	
Combined other	177/1651 (10.7%)	1.098 (0.923-1.305)	
Malignant disease			
No malignancy	665/6435 (10.3%)	Ref.	P=0.015
Gynecological malignancy local	67/899 (7.5%)	0.709 (0.552-0.912)	
Gynecological malignancy locally advanced	56/501 (11.2%)	1.082 (0.824-1.421)	
Gynecological malignancy peritoneal metastasized	3/52 (3.9%)	0.549 (0.177 -1.706)	
Malignancy other origin	21/145 (14.5%)	1.472 (0.953-2.273)	
Intra-abdominal infection			
No	792/8003 (9.9%)	Ref.	
Yes	20/29 (69.0%)	9.320 (5.978-14.531)	P<0.001
History of radiotherapy			
No	808/8021 (10.1%)	Ref.	
Yes	4/11 (36.4%)	4.310 (1.614-11.511)	P=0.004
Mesh placement			
No	731/7923 (9.2%)	Ref.	
Yes	81/109 (74.3%)	12.158 (9.653-15.313)	P<0.001
IBD			
No	807/8015 (10.7%)	Ref.	
Yes	5/17 (29.4%)	3.221 (1.337-7.759)	P=0.009
Endometriosis			
No	725/7171 (10.1%)	Ref.	
Yes	87/861 (10.1%)	0.989 (0.792-1.235)	P=0.923
Adhesiolysis			
No	791/7741 (10.2%)	Ref.	
Yes	21/291 (7.2%)	0.697 (0.452-1.076)	P=0.103

Table S13: multivariate analysis of reoperation in woman who underwent initial hysterectomy.

	N/N total (%)	HR (95% CI)	Sig.
Approach			
Open	487/5594 (8.7%)	1.006 (0.670-1.590)	p<0.001
Laparoscopic	25/313 (8.0%)	Ref.	
Transvaginal	300/2125 (14.1%)	2.199 (1.443-3.352)	
Age		0.987 (0.981-0.994)	p<0.001
Operation site			
Uterus	170/1673 (10.2%)	0.984 (0.815-1.188)	P=0.626
Combined gynecologic	465/4708 (9.9%)	Ref.	
Combined other	177/1651 (10.7%)	1.108 (0.887-1.385)	
Malignant disease			
No malignancy	665/6435 (10.3%)	Ref.	P=0.224
Gynecological malignancy local	67/899 (7.5%)	0.961 (0.733-1.260)	
Gynecological malignancy locally advanced	56/501 (11.2%)	1.189 (0.855-1.653)	
Gynecological malignancy peritoneal metastasized	3/52 (3.9%)	0.657 (0.209-2.068)	
Malignancy other origin	21/145 (14.5%)	1.659 (1.032-2.667)	
Intra-abdominal infection			
No	792/8003 (9.9%)	Ref.	
Yes	20/29 (69.0%)	7.277 (4.622-11.456)	p<0.001
History of radiotherapy			
No	808/8021 (10.1%)	Ref.	
Yes	4/11 (36.4%)	6.486 (2.408-17.471)	p<0.001
Mesh placement			
No	731/7923 (9.2%)	Ref.	
Yes	81/109 (74.3%)	13.148 (10.311-16.765)	P<0.001
IBD			
No	807/8015 (10.7%)	Ref.	
Yes	5/17 (29.4%)	1.711 (0.688-4.255)	P=0.248

Figure S1: Nomogram to predict readmission directly related to adhesions in woman who underwent initial hysterectomy.

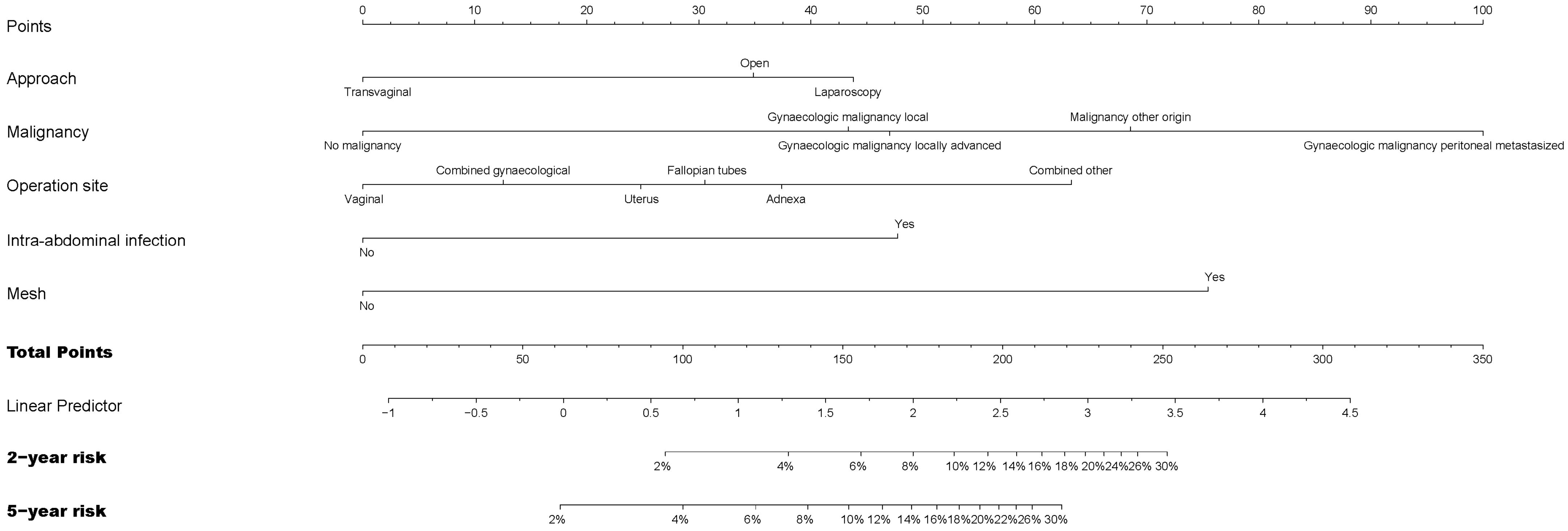

Figure S2: Nomogram to predict readmission possibly related to adhesions in woman who underwent initial hysterectomy.

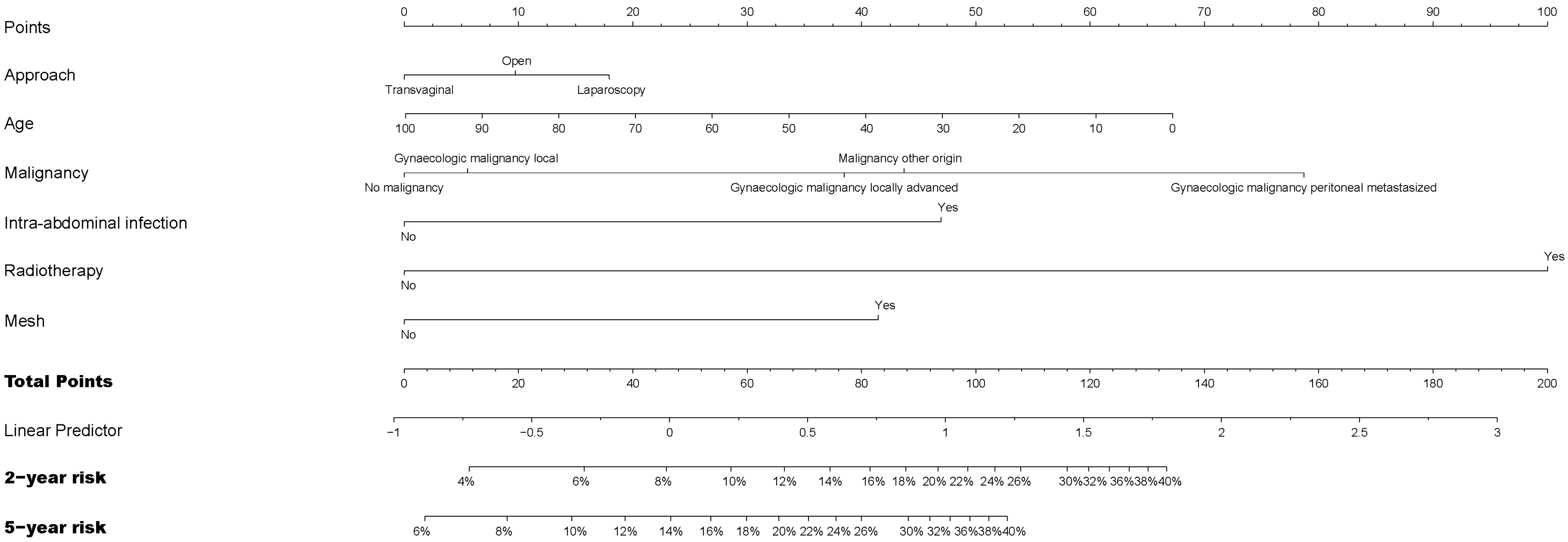

Figure S3: Nomogram to predict reoperation in woman who underwent initial hysterectomy.

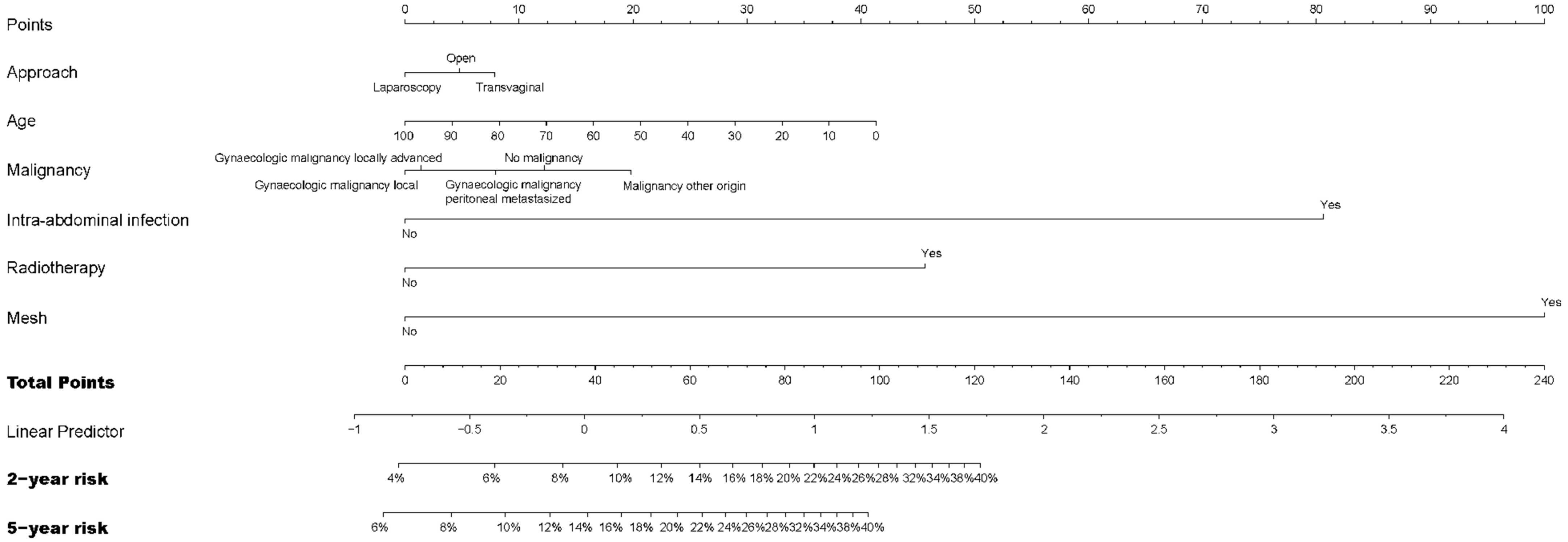

Supplement: Supplementary file 1 [file jcm-12-01351-s001.zip › jcm-2183408-supplementary.pdf]
